# Utilization of Natural Zeolite and Perlite as Landfill Liners for *in Situ* Leachate Treatment in Landfills

**DOI:** 10.3390/ijerph9051581

**Published:** 2012-05-03

**Authors:** Ummukulsum Ozel, Andaç Akdemir, Osman Nuri Ergun

**Affiliations:** 1 Department of Civil Engineering, Karadeniz Technical University, Trabzon 61080, Turkey; 2 Department of Environmental Engineering, Ondokuz Mayis University, Samsun 55139, Turkey; Email: aakdemir@omu.edu.tr (A.A.); onergun55@gmail.com (O.N.E.)

**Keywords:** landfill liner, leachate treatment, natural zeolite, perlite, bentonite

## Abstract

The potential long term environmental impacts of a landfill on groundwater quality depend on its liner material properties. In case synthetic liner materials are damaged during the construction or operation, many of the original chemical and biological constituents are removed by filtration and the adsorptive action of natural liner materials such as natural zeolite, perlite and bentonite minerals. Before leachate treatment, reduction of these constituents is important not only to leachate percolation, but also treatment cost and efficiency. In this study, the pollutant removal efficiency from the leachate was investigated for natural natural zeolite, expanded perlite and bentonite. Experimental studies was performed in boxes made of glass and with 1:10 sloping. Leachate quantity was determined and pH, electrical conductivity (EC), nitrate (NO_3_-N), ammonium-nitrogen (NH_4_-N), phosphate (PO_4_), chemical oxygen demand (COD) and organic matter in leachate samples were measured and the measurement was compared with control process (System 4). The results showed that natural zeolite was effective in removing NO_3_, NH_4_, PO_4_, COD and organic matter with removal efficiencies of 91.20, 95.6, 95.5, 83.4 and 87.8%, respectively. Expanded perlite has high efficiency removing of NO_3_, PO_4_ and COD 83.2, 91.0 and 62.5%, respectively, but it was unsuccessful in reducing NH_4_ (1.5%).

## 1. Introduction

The sanitary landfill plays most important role in the solid waste disposal because it is an economical and final solution. Solid waste leachates with their high organic and inorganic strength and quantities are however major polluting substances compared with wastewaters [1].

Leachate is generated by water passing through solid wastes and biological and chemical constituents leaching into the subsoil [2,3]. Leachate discharged into the subsoil causes groundwater pollution, so landfill technology needs to focus on preventing and controlling leachate problems. Therefore, barrier systems are used in order to mitigate the negative effects of the leachate.

Barrier systems (*liner systems*) which are made both natural and synthetic materials are used as the base and sloping sides of landfill in order to isolate to leachate from waste sites [4,5]. There are several different types of liner systems, such as single liner systems, composite liner systems, double liner systems, and multiple liner systems based on combinations of liner materials and liquid collection layers to contain and collect the leachate and landfill gas [6].

As leachate percolates through the layer, it is important that these systems have a high potential for retaining toxic materials by adsorption, precipitation, and/or redox processes. In case liner materials are damaged during the construction or operation, many of original chemical and biological constituents can be removed by the filtration and adsorptive action of natural liner materials [2,7,8], so clay rocks, clay mineral admixtures, and zeolite admixtures are widely used as hydraulic barriers underneath waste containment systems. These materials are also used as constituents of *in situ* geological barriers (*permeable treatment barriers*), *i.e*., waste deposit locations [5,8,9].

In recent years, due to their high hydraulic conductivity natural zeolites having cation exchange capacity (CEC) between 200 and 400 meq/100 g have been used along with clay minerals such as kaolinite (15 meq/100 g), illite (40 meq/100 g), bentonite (60 meq/100 g) [10]. In addition to these, perlite also is commonly used for wastewater treatment. The aim of this study was to remove some pollutants in landfill leachate as an *in situ* treatment taking advantage of thr adsorption properties of the natural sealing materials. 

## 2. Materials and Methods

### 2.1. Properties of the Materials

#### 2.1.1. Natural Zeolite

Zeolites are crystalline aluminosilicates of alkali or alkali earth elements, such as sodium, potassium, and calcium [11] and possess three dimensional frameworks of SiO_4_ and AlO_4_ tetrahedra linked by shared oxygen [12]. The mineral framework contains openings and internal voids or channels of fixed dimensions characteristic of the individual varieties. These internal channels are occupied by leaving water (full 50% of void volume water) [12].

In this study, natural zeolite samples were obtained after crushing from Manisa-Gordes in Turkey. They were sorted into −16 + 35 grain size by sieving in the laboratory. It is indicated that the analyzed natural zeolite-rich tuff contain 95% klinoptilolit (Ca Si_7_ Al_2_O_18_.6H_2_O), 5% heulandite (K Na_2_ Ca_2_ Si_2_ 9Al_7_ O_72_ 4H_2_O). The chemical analysis results of the natural zeolite were confirmed by X-ray fluorescence data that is shown that in Table 1.

**Table 1:** Results of chemical analysis of the bentonite, natural zeolite and expanded perlite used in experiment.

Elemental Oxide	Weight %		
	Bentonite	Natural Zeolite	Expanded Perlite
Na_2_O	1.8	0.40	3.29
MgO	-	1.40	0.18
Al_2_O_3_	17	11.80	11.90
SiO_2_	61	71.00	72.90
P_2_O_5_	-	-	0.02
CaO	2.5	3.40	0.79
TiO_2_	-	0.10	-
MnO	4	-	0.05
K_2_O	0.5	2.40	4.47
F_2_O_3_	3	1.70	0.53
SO_3_	-	0.12	-
Loss on ignition(LOI)	6	6.87	1.00

#### 2.1.2. Expanded Perlite

Perlite is a hydrated volcanic glass composed chiefly of amorphous silica with 12–18% aluminum oxide, minus the oxides of potassium and sodium, and with small amounts of iron, magnesium, calcium, and titanium [12]. As most perlites have a high silica content, usually greater than 70%, and are adsorptive, they are chemically inert in many environments and hence are excellent filter aids and fillers used in various processes and materials [13].

Because of the 2–5% water content, this rock is commercially valuable and most of the perlite used commercially is in expanded form. Upon heating above 870 °C contained water is removed, low density particles with cellular interiors are formed. These particles are used due to their chemical inertness, physical resilience and water retention ability [12].

Expanded perlite samples produced at the Izmir-Bergama region of Turkey were obtained from the Akper Minning Company and reduced to −16 + 35 grain size by sieving in the laboratory. Chemical analysis results of the samples confirmed by X-ray fluorescence data are shown in Table 1. Its bulk density is 80–120 kg/m^3^. The moisture content is 0 11%.

#### 2.1.3. Bentonite

Bentonite is a clay consisting essentially of the mineral smectite of the montmorillonite group, which has Na^+^ and Ca^2+^ end members [14] Montmorillonite is composed of a central alumina octahedral layer sandwiched between tetrahedral silica layers [12]. Bentonite deposits are mainly formed by alteration of volcanic rock or by direct precipitation (authigenesis) in alkaline continental basins [15]. Deposits of bentonite can be found on almost all continents; however, the features and the properties of the material differ greatly from zone to zone. It has the special feature of swelling when in contact with water, constituting a tixotropic gel [14]. Although it is insoluble in water, it swells to approximately twelve times its volume when added to water and does not swell in organic solvents including absolute alcohol, isopropanol, glycerin and fixed oils [12].

Bentonites are classified into two groups: Na-bentonite and Ca-bentonite. The swelling properties of Na bentonites are reater than those of Ca-bentonites and Na bentonites are preferred in landfills because they have low shrinkage and hydraulic conductivity [16,17]. In this study, the −16 + 35 grain size bentonite samples which were used were prepared by sieving in the laboratory and were obtained from a bentonite mine in Edirne. The chemical analysis results of the natural bentonites were confirmed by X-ray fluorescence and are shown in Table 1.

### 2.2. Obtaining and Preparing of the Solid Wastes Sample to Experiments

Solid wastes were used in the cabins were provided from containers in the -Kurupelit region of Turkey and they were collected as mixed wastes. After their organic part was separated, they were mixed until homogeneous and then divided into four groups. These were mixed and divided again into four groups. This operation was repeated until four group of 20 kg each were obtained. Thus the organic wastes added to the cabins were identical. Physical composition of the waste mixture is shown in Table 2.

**Table 2:** Physical composition of the solid waste samples [18].

Composition	Weight %
Organic Waste	80.6
Paper-Cartoon	6.10
Nylon-Plastic	8.06
Metal	2.01
Glass	3.23

The aim of this study was to investigate the effects of natural zeolite, expanded perlite and bentonite when used as alternate liner materials in a sanitary landfill on the transmission of leachate and treatment of pollutants in the leachate. Natural materials was placed as the base of cabins is made of glass with 1:10 slope; in this study the solid waste was added on top of the natural materials. No material was placed into one cabin base in order to compare with the chemical characteristic of the leachate for the removal of each material.

**Figure 1:**
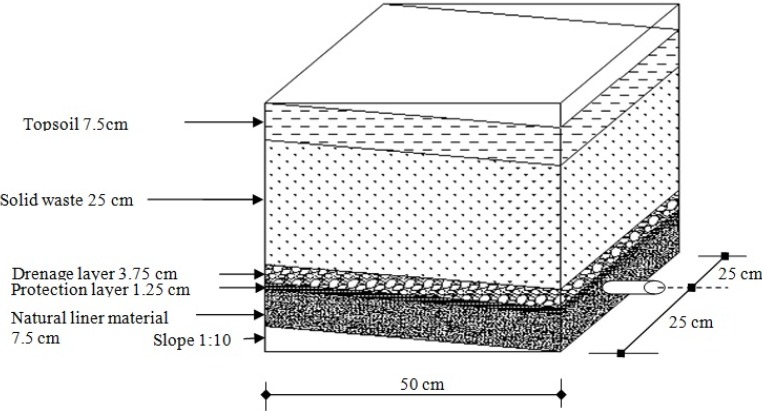
Schematic of the test apparatus.

Contents of the laboratory scale cabins were prepared in agreement with guidelines of the Turkish Ministry of Environment and Forestry (MEF). A schematic of the test apparatus is illustrated in Figure 1 and their component rates are listed in Table 3. Experimental systems were left open to atmosphere in order to imitate the characteristics of real landfills. The region’s meteorological data was obtained from the Samsun Meteorology Regional Directorate. 

Landfill leachate samples were analyzed for periods of one week and two weeks. The leachate quantity, pH, electrical conductivity (EC), nitrate (NO_3_-N), ammonium-nitrogen (NH_4_-N), orthophosphate (PO_4_), chemical oxygen demand (COD) and organic matter parameter measurements were performed. The pH and EC were measured using a digital pH meter and conductometer (Cyberscan pH 510 meter, Jenway-4071 ). NO_3_-N, NH_4_-N, PO_4_-P and COD were measured in reference to the standard method by using a Thermo Scientific-Aquamate UV-VIS spectrophotometer. Used test methods are equivalent to the corresponding DIN, ISO and APHA.AWWA.WEF methods. Organic matter measurements were performed in reference to the standard method [19].

**Table 3:** Components and their rates in the test apparatus.

System	Components
System 1	Solid Waste (25 cm)
System 2	Natural. Zeolite (7.5 cm) + Sand (1.25 cm) + Gravel (3.75 cm) + Waste (25 cm) + Topsoil (7.5 cm)
System 3	Expanded Perlite (7.5 cm) + Sand (1.25 cm) + Gravel (3.75 cm) + Waste (25 cm) + Topsoil (7.5 cm)
System 4	Bentonite (7.5 cm) + Sand (1.25 cm) + Gravel (3.75 cm) + Waste (25 cm) + Topsoil (7.5 cm)

## 3. Experimental Results

**Figure 2:**
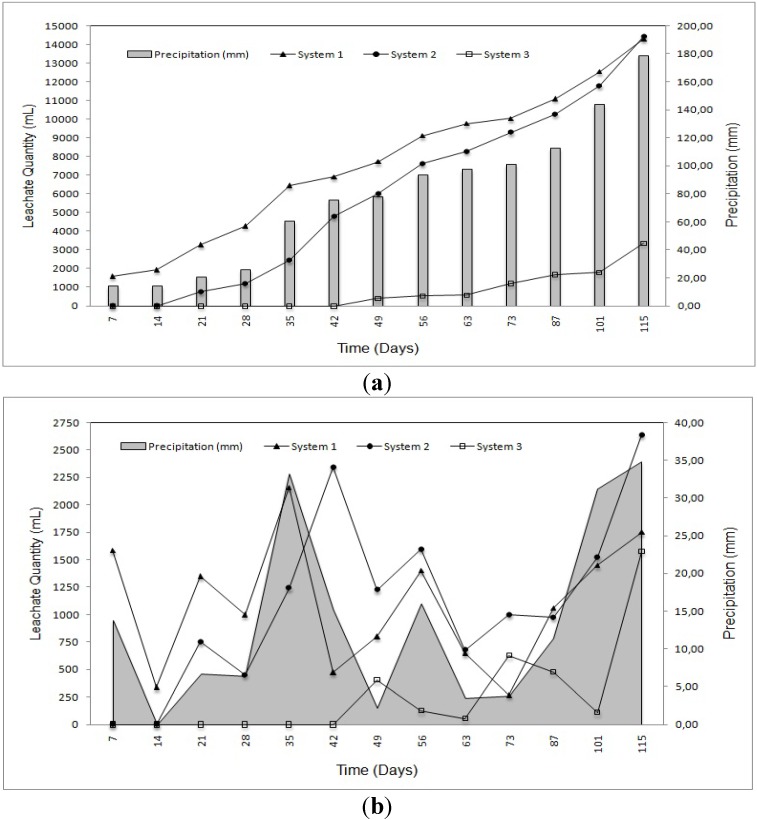
(**a**) Cumulative leachate quantity-precipitation; (**b**) Daily leachate quantity-precipitation.

Cumulative and daily leachate quantities depending on precipitation in the experiments continued during 17 weeks, are shown in Figure 2(a,b). Leachate appeared in the first week and began flowing after the second and seventh week in system 2 and system 3, respectively, but leachate didn’t flow during the experiment in system 4. As a result there are no pollutant measurements for system 4. The chemical characteristics of system 1 are compared with the different landfill leachates in Table 4 and for each system, treatment efficiencies are given in Table 5.

According to the degradation of wastes and seasonal precipitation, the amount of leachate in each system showed an increase (Figure 2(a,b)). However, leachate leaking from system 3 was less than for system 1 and the highest leachate was in system 1 because of the absence of liner material. It is shown that infiltration of leachate for system 2 and system 3 decreased 24.70 and 55.00%, respectively (Table 5).

**Table 4:** Comparison of the leachate characteristics in system 1 with different leachates.

Parameters	pH	EC (μS/cm)	NO_3_-N (mg/L)	NH_4_-N (mg/L)	PO_4_ (mg/L)	COD (mg/L)
System 1	6.73–8.58	6,040–11,750	17.1–40.9	10.5–102.6	63.7–178.9	4,388–9,761
Tchobanoglous ,1993 [2]	6	-	25	1–1,500	20	18,000
Andreottola,1992 [20]	5.3–8.5	-	1.5–50		0.3–25	150–100,000
SWANA, 2004, [21]	6.8	12,000	-	1,180		20,000
SWANA, 2004, [21]	7.2	25,000	-	910		5,600

**Table 5:** Pollutant removal efficiencies in system 2 and system 3.

	Removal Efficiencies (%)	
	System 2	System 3
Leachate quantity	24.70	55.00
NO_3_-N	91.20	83.20
NH_4_-N	95.60	1.50
PO_4_-P	95.50	91.00
COD	83.40	62.50
Organic matter	87.80	48.70

**Figure 3:**
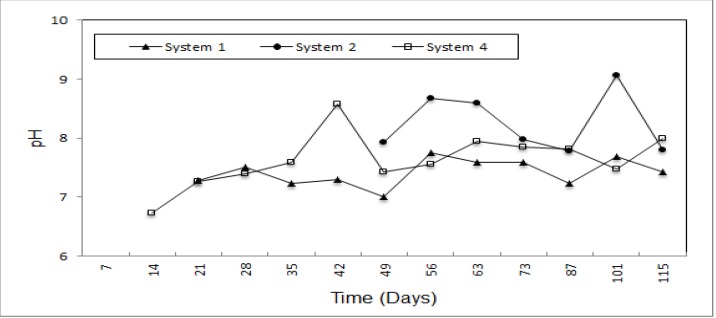
Variation of pH for system 2 and system 3 *versus* system 1.

pH values measured for all systems are also shown in Figure 3. Not only were the pH values of system 2 lower than those of system 1, but they are also higher than system 3. Moreover, pH values in system 3 are the highest among all the systems. In the first phase of the leachate formation, the pH is over 8 and it drops to 6 because of existence of organic acids in the second phase. pH values of system 1 were between 6.73–8.58, and didn’t show any unexpected variation. The highest pH value of system 2 is 7.68, whereas the lowest value of system 3 was measured as 7.78.

**Figure 4:**
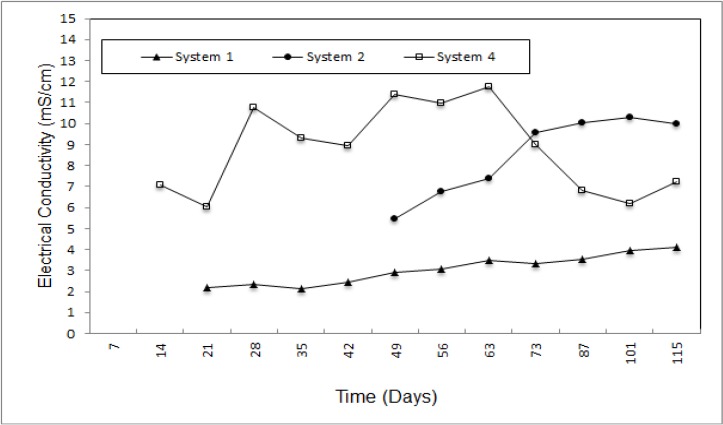
Variation of the leachate electrical conductivity for system 2 and system 3.

SWANA [21] reported that due to organic acids, low pH values caused increased metal solubility and electrical conductivity (EC) that can be used as an indicator of the abundance of these dissolved inorganic species or total concentration of ions [22]. EC values increased *versus* time and the lowest recorded value (6.04 m) was in system 1 (Figure 4). After the EC of system 4 reached the highest value (11.75 mS) it started to reduce. In addition to this, the EC values of system 1 and system 3 increased in during the course of time but system 3 recorded higher values than system 2. The EC values of system 2 and system 3 varied between 2.16–4.09 mS and 5.46–10.3 mS, respectively.

**Figure 5:**
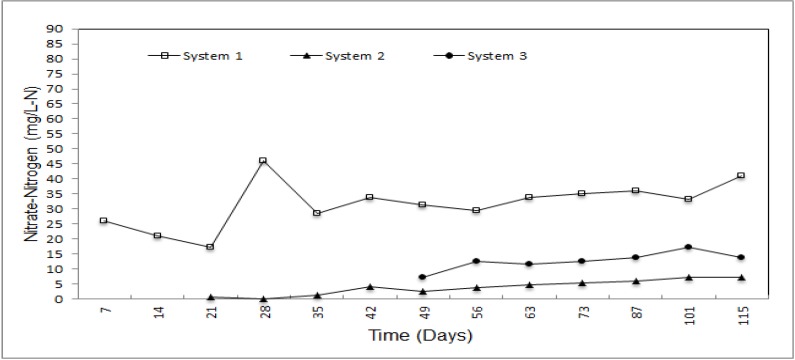
Nitrate-nitrogen concentration of the leachate.

Nitrate concentrations of each system are shown in Figure 5. Increases in nitrate concentrations depending on time were observed in each system and the concentration variations of the different materials are close to straight lines. Nitrate removal of the systems is very high and removal efficiencies are 91.20 and 83.20% for system 2 and 3, respectively. Using natural zeolite as a liner material in landfills is more effective than using expanded perlite. The lowest concentrations of the leachate with respect to natural zeolite and expanded perlite utilization are 2.62 and 7.20 mg/L concurrently and the initial and final concentrations of the nontreated control process are 25.99 and 40.98 mg/L on the 7th and 115th days.

**Figure 6:**
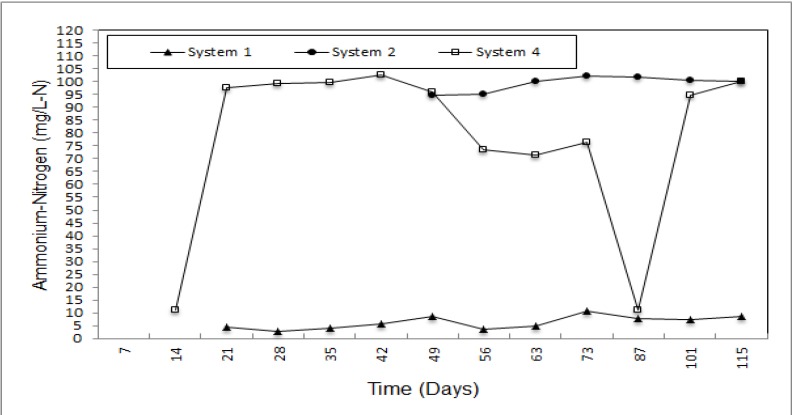
Ammonium-nitrogen concentration of the leachate.

Ammonium-nitrogen (NH_4_-N) concentration was measured during the research and variance of NH_4_-N is given Figure 6. As shown in Figure 6, firstly, the concentration of NH_4_-N for system 1 increased and became fixed and then dropped in. As Johannessen [23] mentioned, high concentrations of NH_4_-N represenrt a third phase for degradation of waste. Although NH_4_-N was observed at low levels in system 2 and its maximum level was measured at 10.54 mg/L-N. The removal efficiency for system 2 was 95.60%. NH_4_-N levels in system 3 were higher than in system 1. Whereas the ammonium concentration decreased in system 1, in system 3 it increased. Therefore treatment performance for system 3 was lower than for system 2.

**Figure 7:**
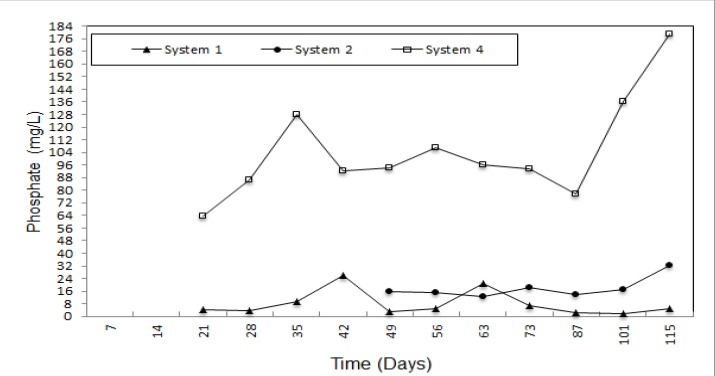
Phosphate concentration of the leachate.

According to Figure 7, the phosphate concentration of leachate for system 2 and system 3 was lower than in system 1 and changed *versus* time. Variable values from 178.93 mg/L to 63.71 mg/L, in system 1 were reduced by using natural zeolite and expanded perlite. The phosphate removal efficiencies were 95.50% and 91.0% for system 2 and system 3, respectively.

**Figure 8:**
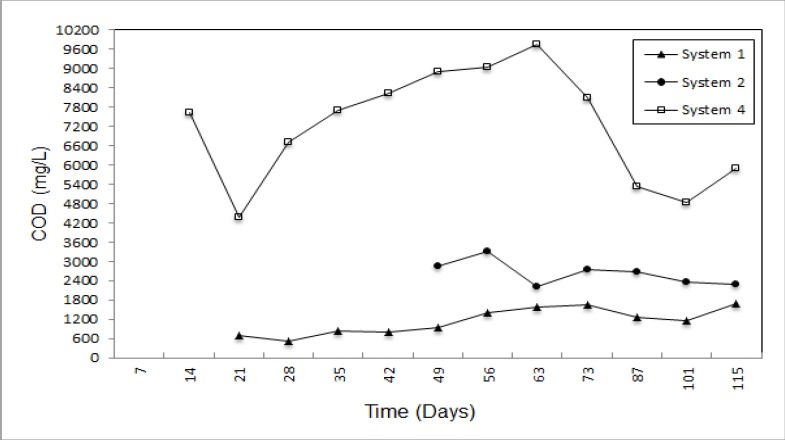
COD concentration of leachate.

Tchobanoglous [2] reported that typical value of chemical oxygen demand (COD) is 18,000 mg/L and as shown in Figure 8, the highest value of system 1 reached 9,761.50 mg/L. However using natural zeolite and expanded perlite reduced this to 1,138.10 mg/L and 2,633.62 mg/L on average. By this means removal efficiency is 83.40 and 62.50% for natural zeolite and expanded perlite, respectively. 

**Figure 9:**
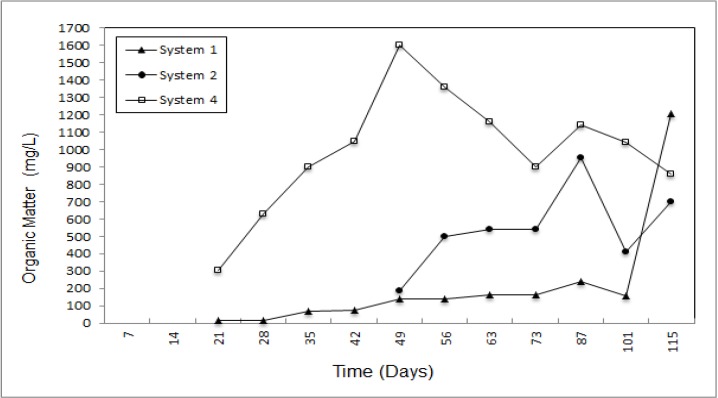
Organic matter concentration of the leachate.

The amount of organic matter builds up over time and the increasing organic matters in leachate originate organic acids. In system 4, it was reduced by means of a reduction of the decomposition rate and its maximum concentration reached from 340 mg/L to 1,600 mg/L. According to Figure 9, natural zeolite is more effective than expanded perlite and removal efficiency was 87.8% and 48.7% for system 2 and 3, respectively.

## 4. Conclusions

Natural zeolite and expanded perlite did not differ much for the removal of NO_3_-N, PO_4_-P and COD, whose removal percentages were determined to be 91.20 and 83.20, 95.50 and 91.00 and 83.40 and 62.50, respectively. Moreover, natural zeolite achieved effective removal of NH_4_-N and organic matter (91.20 and 87.80 %, respectively). Although natural zeolite was not effective reducing leachate quantity, natural zeolite and expanded perlite show good performance for *in-situ* leachate treatment. On the other hand, due to the swelling feature of bentonite when it contacts water, no leachate was obtained from system 4. If natural zeolite and expanded perlite is used together in different proportions, both accumulated leachate quantity and removal of NH_4_-N, COD and organic matter can be increased. In addition this, the swelling featuree of bentonite can be used to fill up the spaces between of natural zeolite and expanded perlite particles. Even if the rate of filling with bentonite is low, it will serve to both decrease both percolation of leachate and increase the removal efficiency of pollutants.
